# MicroRNAs and Their Associated Genes Regulating the Acrosome Reaction in Sperm of High- versus Low-Fertility Holstein Bulls

**DOI:** 10.3390/ani14060833

**Published:** 2024-03-08

**Authors:** Vanmathy Kasimanickam, Ramanathan Kasimanickam

**Affiliations:** 1 Center for Reproductive Biology, College of Veterinary Medicine, Washington State University, Pullman, WA 99164, USA; vkasiman@wsu.edu; 2 AARVEE Animal Biotech LLC, Corvallis, OR 97333, USA; 3 Department of Veterinary Clinical Sciences, College of Veterinary Medicine, Washington State University, Pullman, WA 99164, USA

**Keywords:** dairy bulls, fertility, spermatozoa, acrosome reaction, microRNA, mRNA, bioinformatics

## Abstract

**Simple Summary:**

The objective was to identify candidate miRNAs and their integrated genes regulating acrosome function in capacitated sperm of high- versus low-fertility dairy bulls and to elucidate functional biological pathways using a systems biology approach featuring miRNA–mRNA cluster analyses. Based on categorized bovine miRNAs (*n* = 84), 19 were differentially expressed in high- compared to low-fertility capacitated sperm (*p* ≤ 0.05, fold regulation ≥ 2 magnitudes). mRNA expression of highly scored integrated genes of differentially expressed miRNAs was greater, ranging from 2.0 to 9.1-fold (*p* < 0.05) in high- compared to low-fertility sperm, with predicted pathways regulating acrosome vesicle exocytosis, acrosome reaction, and binding of sperm to zona pellucida. In conclusion, highly differentially expressed miRNAs in high-fertility bovine sperm regulating acrosome function have potential for predicting bull fertility.

**Abstract:**

Bioinformatics envisage experimental data as illustrated biological networks, exploring roles of individual proteins and their interactions with other proteins in regulation of biological functions. The objective was to identify differentially expressed miRNAs and their associated genes regulating the acrosome reaction in capacitated sperm of high- compared to low-fertility dairy bulls and to elucidate biological functional pathways using a systems biology approach, featuring miRNA–mRNA cluster analysis. Categorized bovine-specific miRNAs (*n* = 84) were analyzed by RT-PCR; 19 were differentially expressed in high- compared to low-fertility sperm (*p* ≤ 0.05, fold regulation ≥ 2 magnitudes). Six miRNAs (bta-miR-129-5p, bta-miR-193a-3p, bta-miR-217, bta-mir-296-5p, bta-miR-27a, and bta-miR-320a) were highly upregulated (*p* < 0.05; fold regulation ≥ 5 magnitudes) in high- compared to low-fertility sperm. Highly scored integrated genes of differentially expressed miRNAs predicted associations with pathways regulating acrosome vesicle exocytosis, acrosome reaction, and sperm-oocyte binding. The mRNA expressions of genes associated with the acrosome reaction (including hub genes) were greater, ranging from 2.0 to 9.1-fold (*p* < 0.05) in high- compared to low-fertility capacitated bull sperm. In conclusion, differentially expressed miRNAs in high-fertility bovine sperm regulating acrosome functions have potential for predicting bull fertility.

## 1. Introduction

Sperm carry the paternal genome and a wide catalog of molecules including RNAs with roles in fertilization and embryogenesis. Spermatogenesis, the process in which germ cells are produced and progress into mature sperm, is controlled by multiple factors. DNA polymorphisms and gene expression have been linked to sperm quality and/or fertility in several mammalian species, including cattle [1].

High-quality sperm is pivotal to enhance propagation of the best genetic material in cattle. However, bull replacement due to insufficient sperm quality remains an economic hurdle [2]. Consequently, ejaculated sperm is subjected to quality control. To determine ejaculate quality and predict fertilizing ability, bulls are regularly evaluated by measuring sperm structural and functional traits including concentration, motility, viability, and morphology [3,4,5]. Heritability of these traits is low to moderate.

Molecular mechanisms controlling sperm quality are not fully understood [6,7,8,9]. Few genetic or transcriptomic studies have used high-throughput techniques to investigate the genetic basis of sperm. The presence of RNA molecules in the sperm is well documented, and sperm RNAs and their gene abundances are mostly associated with prior transcriptional events linked to spermatogenesis, fertility, and embryo development [10,11]. A complex suite of RNAs is present in sperm, including coding (mRNA), long noncoding RNAs (e.g., circular RNA–circRNA) and short noncoding RNAs (e.g., microRNA–miRNA or Piwi interacting RNA–piRNA) [12]. There are associations between RNA abundances and semen quality in mammals [13,14], but further research is needed to explore their mechanisms. Genetic complexity contributing to sperm quality involves several molecular mechanisms and pathways that are highly interconnected [15]. Complex traits are quantitative traits that do not follow simple Mendelian inheritance laws; these traits are typically affected by numerous genomic regions, many of which may explain only a small proportion of the phenotypic variance in genome-wide association studies or differential expression analyses [16].

The acrosome is a membrane-limited granular secretory vesicle that occupies the anterior part of the sperm nucleus and comprises numerous acid hydrolytic enzymes. Acrosome reaction (AR), or acrosome exocytosis, is a synchronized and tightly regulated process resulting in a final structural modification of sperm in the female reproductive tract that is vital for fertilization [17,18]. Acrosome exocytosis is a chronological process including the opening of hundreds of fusion pores between the outer acrosomal membrane and sperm plasma membrane. It occurs before sperm penetration of the extracellular layer of the zona pellucida (ZP), mediated by a homologous mechanism in which sperm use the synaptosome-associated protein receptor (SNARE) fusion machinery and regulatory components [19,20,21].

Acrosome integrity is vital for fertilization; high-fertility bulls have a large proportion of sperm with an intact acrosome. The ability of sperm to release acrosomal contents (AR) in a timely manner in response to proper stimuli is critical for successful fertilization [22]. Although the AR is a precise, well-regulated exocytotic process, spontaneous AR can occur, and ejaculates with high numbers of spontaneous acrosome-reacted sperm reduce fertilization rates [23,24,25]. Great progress has been achieved in elucidating morphological and molecular changes of acrosome exocytosis. Nevertheless, whether additional molecular elements can modulate this process remains unclear. Thus, determining acrosome reacting ability and the associated molecular mechanism is critical to elucidate contributions to male fertility.

Our aim was to identify differentially expressed miRNAs and their associated genes regulating the AR in capacitated sperm of high- versus low-fertility dairy bulls, and to elucidate biological functional pathways using a systems biology approach featuring miRNA–mRNA cluster analysis.

## 2. Materials and Methods

### 2.1. Ethics Statement

This study was exempt from the review of the Institutional Animal Care and Use Committee of Washington State University (Tissue Use ASAF 4059).

### 2.2. Bulls and Semen Sample Processing

Holstein (2.2 ± 0.06 years old) bulls were selected based on sire conception rate (SCR) estimates (≥500 services), with 10 bulls each in the low- (≤2 SCR) and high-fertility (≥2 SCR) groups included. The semen samples were collected during the month of August at room temperature in a USDA-approved semen collection facility. From each bull, two ejaculates were collected via artificial vagina, combined into a single batch, transported to the laboratory for processing, loaded in 0.5 mL French straws, and cryopreserved in liquid nitrogen.

Capacitation was induced as described [26,27,28]. Briefly, frozen thawed sperm from each sire were incubated (4 h at 38.5 °C under an atmosphere of 5% CO_2_, 95% air, and 100% humidity) at a concentration of 20 × 10^6^ sperm/mL under capacitation-supporting conditions in capacitation medium (100 mM NaCl, 3.1 mM KCl, 1.5 mM MgCl_2_, 25 mM NaHCO_3_, 0.29 mM KH_2_PO_4_, 21.6 mM sodium lactate, 0.1 mM sodium pyruvate, 2 mM CaCl_2_, 20 mM HEPES [pH 7.4], 50 mg/mL BSA, 10 U/mL penicillin, and 20 mg/mL heparin).

### 2.3. Sperm Mature miRNA Profiling Using Real-Time PCR

Total RNA was isolated from sperm using QIAzol reagent (Qiagen, Valencia, CA, USA). For miRNA analysis, 250 ng of total RNA was reverse-transcribed to cDNA using a miScript II RT kit (Qiagen) [29]. Quantitative RT-PCR was performed using miScript miRNA PCR arrays in combination with the miScript SYBR Green PCR Kit (miScript Universal reverse primer and QuantiTect SYBR Green PCR Master Mix) on a StepOnePlus cycler (Applied Biosystems, Foster City, CA, USA). Relative miRNA expression was normalized to U6 small nuclear RNA (snRNA). Three replicates were used for each sample.

Elucidation of DE-miRNAs was accomplished using 84 bovine mature miRNAs (Table S1). These miRNAs were selected from the miRNA genome database. Data quality control was examined to assess amplification reproducibility and reverse transcription efficiency, and to detect any other contamination in amplified samples [30,31]. The control used was cel-miR-39-3p. In addition, two internal normalizers, two reverse transcription controls, and two positive controls were included to ensure efficiency of the array, reagents, and instrument. Distributions of CT values and raw data averages were reviewed in both groups. Mean CT values were converted into linear 2ΔΔCT values and *p* values were calculated with a Student’s *t*-test (SAS 9.4 for Windows, SAS Institute, Cary, NC, USA), with *p* ≤ 0.05 considered significant.

### 2.4. Bioinformatics Analysis

#### 2.4.1. Conserved Nucleotide Sequences

Differentially expressed bovine miRNA nucleotide sequences were compared to human miRNAs for similarity using a miRNA database (www.mirbase.org, accessed on 23 June 2023) [32,33].

#### 2.4.2. Identification of Target and Predicted Genes of Differentially Expressed miRNAs

Associated genes of DE-miRNAs were envisaged using miRNet (http://www.mirnet.ca/, accessed on 23 June 2023) [34], integrating three databases (TarBase, miRTarBase, and miRecords).

#### 2.4.3. Construction of Protein–Protein Interaction Network and Screening of Hub Genes

Protein–protein interaction (PPI) networks of DE-miRNAs’ predicted target genes were elucidated using the Search Tool for the Retrieval of Interacting Genes/Proteins (STRING) online database (http://stringdb.org/, accessed on 23 June 2023) [35]. Further, Gene Ontology (GO) functional annotation for biological process and Kyoto Encyclopedia of Genes and Genomes (KEGG) pathway enrichment analysis were employed. For all statistical analyses, *p* < 0.05 was regarded as significant. The PPI interaction was exported to Cytoscape software (Version 3.9) and visualized [36]. The top 20 hub genes were selected using the Maximal Clique Centrality (MCC) method [37]. Cluster analysis was performed using ClueGO (http://www.ici.upmc.fr/cluego.shtml, accessed on 23 June 2023) to assimilate GO terms as well as KEGG pathways (k score = 3) [38].

### 2.5. Monitoring Dynamics of Sperm Acrosome Membrane

Acrosome reaction (%) was determined for cryopreserved, capacitated, and acrosome reaction-induced sperm populations.

#### Sperm Capacitation and Acrosome Induction

Sperm capacitation was induced using calcium ionophore A23187 (Molecular Probes) as described [39,40,41], following incubation under 5% CO_2_, 95% air, and 100% humidity for 6 min [41]. The reaction was stopped by adding 100 μL of 70% (*v*/*v*) ethanol.

Acrosome-reacted sperm were identified by the acrosomal marker FITC-conjugated peanut agglutinin (Sigma–Aldrich, St. Louis, MO, USA) staining method [28], with 400 sperm counted at ×400 magnification under fluorescence microscopy (Leitz, Laborlux S). Percentages of acrosome-intact and -reacted sperm were determined for each sample in triplicates.

Intact acrosome percentages were compared among control, capacitated, and acrosome-induced sperm populations between high- and low-fertility bulls by ANOVA (SAS 9.4 for Windows).

### 2.6. Determination of Sperm mRNA Expressions of Sperm Acrosome Genes by Real-Time Polymerase Chain Reaction

Genes such as acrosin (*ACR*), albumin (*ALB*), androgen receptor (*AR*), calmodulin (*CALM*), progestogen-associated endometrial protein (*PAEP*), progesterone receptor (*PGR*), sperm adhesion molecule (*SPAM*), zona pellucida glycoprotein (*ZP*), and glyceraldehyde-3-phosphate dehydrogenase (*GADPH*, housekeeping gene) were selected to substantiate differences in mRNA expressions among cryopreserved, capacitated, and acrosome-induced sperm of high- versus low-fertility bulls.

#### 2.6.1. Total RNA Extraction from Sperm

Three sperm populations, namely cryopreserved, capacitated, and acrosome-induced sperm from high- and low-fertility bulls were used. Semen samples (100 × 10^6^ sperm) were diluted in PBS (pH = 7.4) at room temperature and centrifuged at 1000× *g* for ~10 min (low brake speed and room temperature). Sperm pellets were resuspended in PBS and centrifuged again using the same conditions (three washing steps). Total RNA extraction and cDNA preparation methods were as described in Section 2.3.

#### 2.6.2. Polymerase Chain Reaction of Genes of Interest

For quantitative RT-PCR analysis of mRNA, 500 ng of total RNA was reverse-transcribed to complementary DNA (cDNA) using iScript cDNA Synthesis kit (Bio-Rad, Hercules, CA, USA). Quantitative RT-PCR was performed using a SYBR Green PCR kit on StepOne Plus thermocycler (Applied Biosystems Inc., Waltham, MA, USA). Analysis of gene expression was performed using the 2DDCT method, and relative gene expression was normalized to GAPDH mRNA. Three replicates were measured for each sample. Primer sequences are listed in Table S2.

#### 2.6.3. Statistical Analyses to Determine Differences in mRNA Expression

The RT-PCR data were analyzed by Student’s *t*-test (SAS 9.4 for Windows), using 2-DDCt values to ascertain significance of differences in mRNA expressions of cryopreserved, capacitated, and acrosome-induced sperm populations between high- and low-fertility bulls. In addition, the RT-PCR data were analyzed by Student’s *t*-test using 2-DDCt values to ascertain statistical significance of any differences among cryopreserved, capacitated, and acrosome- induced sperm populations in high-fertility bulls. For all statistical analyses, *p* ≤ 0.05 was considered significant.

## 3. Results

### 3.1. miRNA Expression between High- and Low-Fertility Bull Sperm

**Figure 1:**
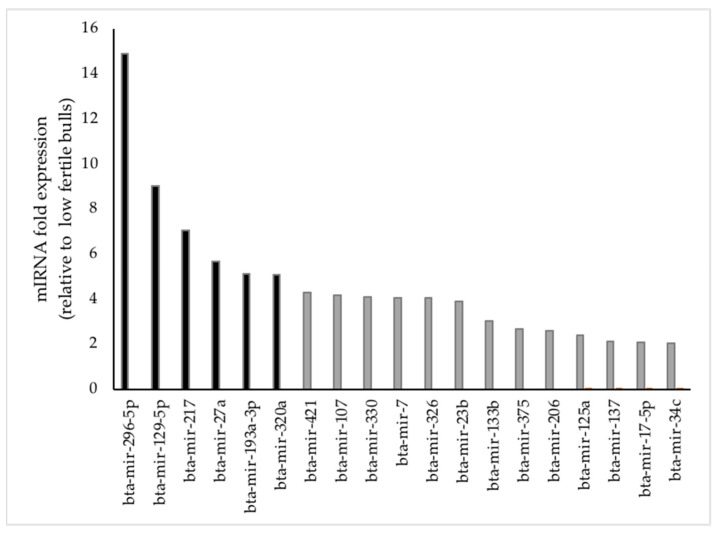
Fold regulation of differentially expressed sperm miRNAs in high- versus low-fertility Holstein bulls. Of 84 bovine-specific well-characterized miRNAs investigated, 19 miRNAs were upregulated (≥2 fold change; *p* < 0.05), whereas six miRNAs were highly upregulated (≥5 fold change; *p* < 0.001) in capacitated sperm of high- versus low-fertility bulls.

Upon semiquantitative profiling of categorized miRNA (*n* = 84) by RT-PCR method, 19 miRNAs were differentially expressed (*p* ≤ 0.05; ≥2 fold expression) in capacitated sperm from high- compared to low-fertility bulls (Figure 1). Among those, six miRNAs (bta-miR-129-5p, bta-miR-193a-3p, bta-miR-217, bta-mir-296-5p, bta-miR-27a, and bta-miR-320a) were highly upregulated (*p* < 0.05; ≥5 fold expression) in high- compared to low-fertility sperm (Figure 1).

### 3.2. Bioinformatics

Bovine DE-miRNA nucleotide sequences were similar to human miRNA nucleotide sequences (Table S3); therefore, human miRNAs were used for further analysis to construct miRNA–mRNA interaction networks and functional enrichment analyses.

The upregulated miRNA and mRNA interaction analysis revealed 960 hits (number of connectivity, degree of miRNA–mRNA interaction) and 15 genes (Supplementary File S1). Further PPI analysis of the 15 predicted genes (55 nodes and 148 edges, PPI enrichment *p* < 1.0 × 10^−16^) revealed 83 enriched gene ontology biological processes (False Recovery Rate, *p* < 0.05) and six KEGG enrichment pathways (False Recovery Rate, *p* < 0.05) (Supplementary File S2). The KEGG pathways were associated with fertilization, interaction with cumulus cells and the zona pellucida, pregnenolone biosynthesis, oocyte meiosis, and calcium signaling.

**Figure 2:**
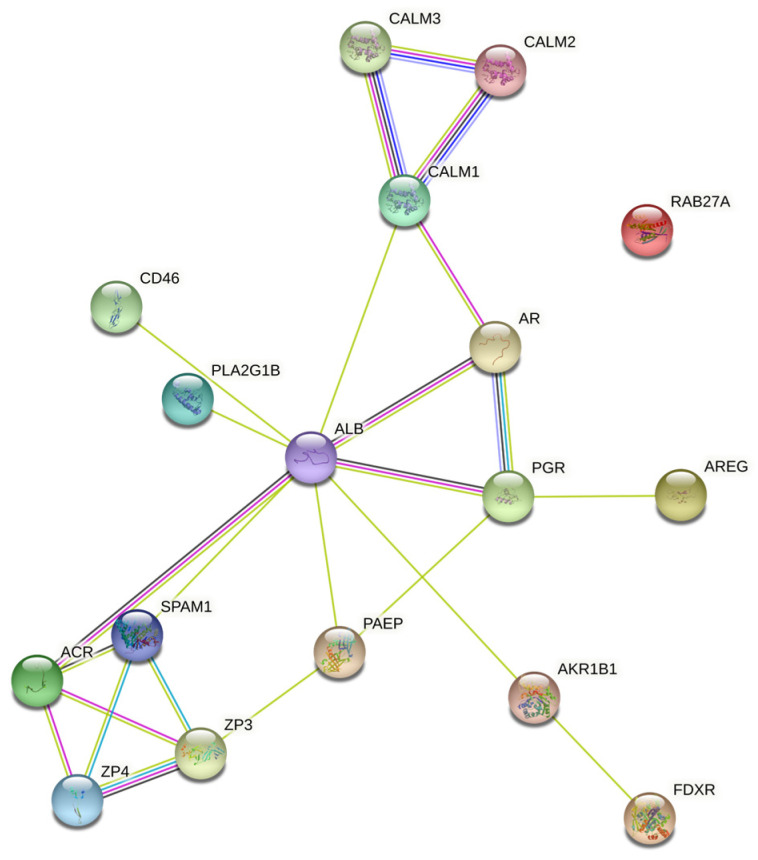
STRING protein–protein interaction (PPI) network. Differentially expressed miRNAs predicted 15 target genes, and their interaction network had 55 nodes and 148 edges (PPI enrichment *p* < 1.0 × 10^−16^). The color nodes represent proteins. The edges (lines) represent interactions.

**Figure 3:**
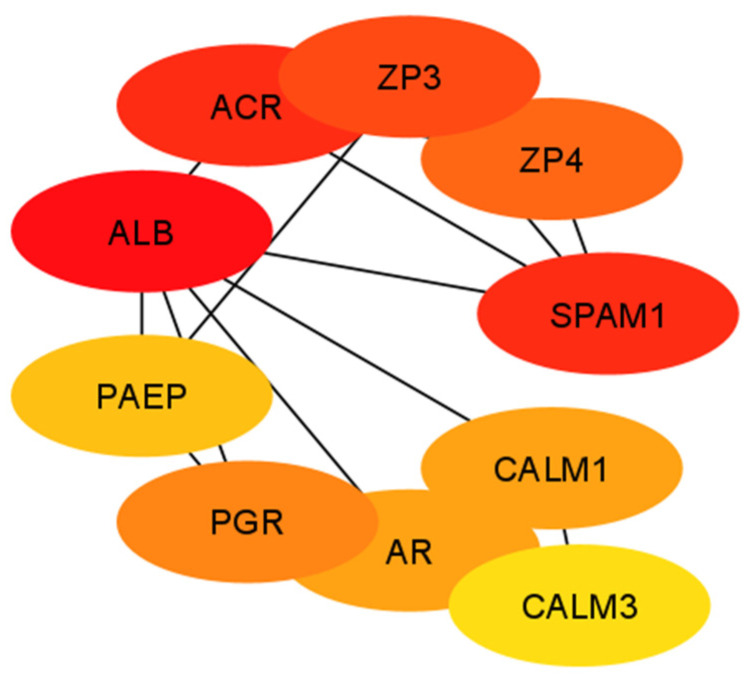
Protein–protein interaction (PPI) network of hub genes of differentially expressed miRNAs. The color gradient from red to yellow denotes a high to a low degree of expression. Black lines indicate interactions between genes.

**Figure 4:**
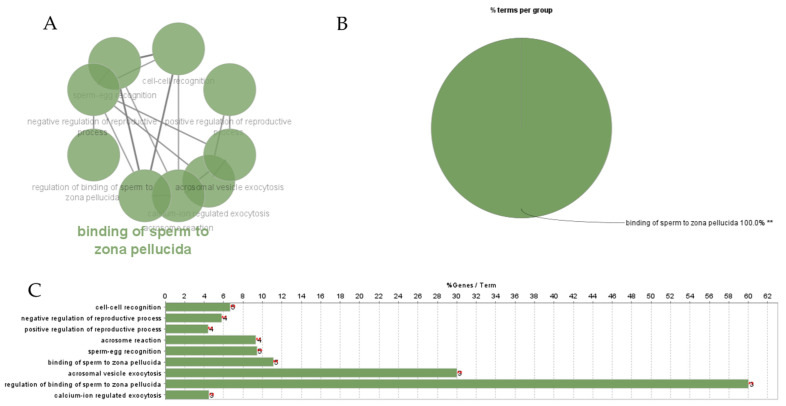
ClueGO analysis of differentially expressed hub genes in highly fertile bulls. (**A**) Functionally grouped network with terms as nodes linked, based on their kappa score levels (≥0.4), where only the label of the most significant term per group is shown. The node size represents the term enrichment significance. Functionally related groups partially overlap. (**B**) Overview chart with functional groups including specific terms for differentially expressed genes. The color gradient shows the gene proportion of each cluster associated with the term. ** *p* < 0.001. (**C**) The bars represent the number of genes associated with the terms. The percentage of genes per term is shown as bar label.

The PPI networks constructed (Figure 2) using the STRING database were imported to Cytoscape software. According to the top- and low-degree essential proteins captured by the Maximal Clique Centrality (MCC) method, the top 10 hubs were selected and are presented in Figure 3. Further cluster network analysis (Supplementary File S3) was performed using ClueGo and results are presented in Figure 4A–C. DE- miRNAs, associated hub genes, and their linked reproductive functions are in Table 1.

### 3.3. Sperm Acrosome Reaction

There were 71 and 68% frozen-thawed sperm with an intact acrosome in high- versus low-fertility bulls, respectively (*p* > 0.1). After induction of capacitation, cryopreserved sperm with an intact acrosome were reduced to 63 and 31%, respectively, and after induction of AR in capacitated sperm, more underwent acrosome reaction in the high- versus low-fertility groups (intact acrosome, 18 vs. 24% for high- vs. low-fertility bulls, respectively, *p* < 0.05).

### 3.4. mRNA Expression of Genes among Capacitated and Acrosome-Induced Sperm Populations in High-Fertility Bulls

**Figure 5:**
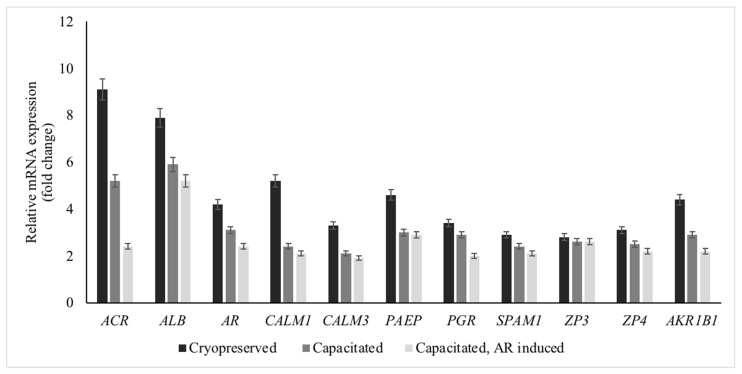
Expression of differentially expressed miRNA predicted genes associated with an AR between high- and low-fertility bulls* and among control (frozen-thawed sperm), capacitated, and AR-induced sperm populations in high-fertility bulls. * Relative mRNA expressions were greater in sperm from high- compared to low-fertility bulls (*p* < 0.05; mRNA relative fold expressions in low-fertility bulls are 1).

mRNA expression of DE-miRNA predicted genes associated with an AR in frozen-thawed, capacitated, and acrosome- induced sperm populations were greater in high- versus low-fertility bulls (Figure 5).

Interestingly, the following pattern of mRNA expression was observed. The mRNA expressions of *ACR*, *AR*, and *AKR1B1* appeared greater in frozen-thawed sperm populations, followed by capacitated sperm populations; the mRNA expression in AR-induced sperm populations was lower compared to the other two sperm populations. However, mRNA expressions of *ALB*, *CALM1*, *CALM3*, and *PAEP* seemed greater in frozen-thawed sperm and capacitated sperm populations compared to the AR-induced sperm population. In addition, mRNA expression of *PGR*, *SPAM1*, and *ZP4* was lower in the AR-induced sperm population compared to the other two sperm populations. However, *SPAM1* and *ZP4* expressions in capacitated sperm populations differed neither from frozen-thawed sperm nor from AR-induced sperm populations, whereas the PGR expression differed between capacitated and AR-induced sperm populations. However, mRNA expression of *ZP3* was similar among those three sperm populations.

## 4. Discussion

The goal was to identify differentially expressed miRNAs in high- versus low-fertility capacitated bull sperm and to use bioinformatics to investigate how top-ranked integrated genes of DE-miRNAs exert wide-ranging connotations related to sperm acrosome function and fertilization. Prioritized miRNAs were examined in capacitated sperm of high- and low-fertility bulls using real-time PCR, eliminating the need for validation after microarray analysis. There were 19 miRNAs at ≥2 fold (*p* ≤ 0.05), with six miRNAs (miR-296-5p, miR-129-5p, miR-217, miR-27a, miR-193a-3p, and miR-320a) at ≥5-fold (*p* < 0.05) that were highly expressed in high- versus low-fertility capacitated bull sperm. These miRNAs were involved in regulation of several sperm functions, including prevention of premature capacitation and promotion of timely capacitation. MicroRNA 296-5p has a conserved binding site in the Pin1 3’-untranslated region (UTR). Interestingly, Pin1 supported advancement of the mitotic cell cycle of spermatogonial stem cells (SSC), required for sperm production from SSCs [75]. The microRNA 320/SERPINA1 axis is involved in regulation of several biological functions, and SERPIN proteins protect sperm from premature capacitation in the epididymis [76]. MicroRNA129-5p participate in modulation of calcium signaling and mitochondrial function and may have important roles in the regulation of capacitation and apoptosis [77,78]. MicroRNA 193-3p promote cell proliferation and inhibit apoptosis and expression of PI3k and p-Akt [79]. They also promote tyrosine phosphorylation during capacitation, possibly through alteration of the PI3K/PDK1/AKT signaling pathway.

In the present study, top integrated genes of the DE-miRNAs’ predicted biological processes demonstrated their critical roles in regulation of sperm function, including AR and zona binding (Table 1). Upregulated miRNA integrated *ACR*, *ALB*, *AREG*, *CALM1*, *CALM2*, *CALM3*, *CD46*, *FDXR*, *PAEP*, *PGR*, *RAB27A*, *SPAM1*, *ZP3*, and *ZP4* genes regulating motility, sperm DNA and mitochondrial function, capacitation, AR and sperm penetration through cumulus cells, and sperm–zona pellucida binding. Further evaluation of mRNA expressions of these genes was performed in cryopreserved, capacitated, and acrosome-induced sperm populations. These genes had greater abundances in high- compared to low-fertility sperm in all three populations.

In domestic animals, ejaculated sperm are unable to fertilize; they must undergo a complex maturation process (capacitation) that allows an AR to occur when they approach or contact an oocyte [80]. Sperm capacitation is a species-specific, time-dependent phenomenon that involves major biochemical and biophysical changes in the sperm membrane. It increases membrane fluidity by elimination of cholesterol from the sperm plasma membrane via sterol acceptors. Capacitation prepares sperm to undergo an AR, a stimulus-induced exocytosis in which hydrolytic enzymes (mostly acrosin) are released by the fusion of the acrosome membrane and the overlying plasma membrane, both of which are essential if fertilization is to progress [81,82]. Ejaculates may exhibit approximately 5 to 20% acrosome-reacted sperm due to spontaneous AR; however, abnormal conditions may induce further AR, decreasing fertilization [83]. Acrosome integrity in ejaculated sperm is crucial for normal fertilization. The AR is a time-dependent phenomenon that cannot take place prematurely or too late. Further, after penetrating through the cumulus oophorous, sperm bind to the zona pellucida. Glycosylation of ZP glycoproteins is important for sperm–ZP interaction. Specific receptors to ZP glycoproteins located over the anterior sperm head facilitate sperm–zona binding [84].

The mRNA expression of each gene (*ACR*, *AKR1B1*, *ALB*, *AR*, *CALM1*, *CALM3*, *PAEP*, *PGR*, *SPAM1*, *ZP3*, and *ZP4)* in cryopreserved, capacitated, and acrosome-induced sperm populations was greater in high- vs. low-fertility bulls. The mRNA expressions of genes were greater in abundance in cryopreserved sperm populations compared to capacitated and acrosome-induced sperm populations, except for *PGR*, *SPAM1*, *ZP3,* and *ZP4* in high-fertility bulls. Further, mRNA expression of *ALB*, *CALM1*, *CALM3*, *PAEP*, *SPAM1*, *ZP3,* and *ZP4* appeared similar among capacitated and acrosome-induced sperm populations in high-fertility bulls. Notably, miR-107 integrated with *ALB*; miR-27a integrated with *PAEP*; miR-34c and miR-449a integrated with *SPAM1*; miR-7 integrated with *ZP3*; and miR-23b integrated with *ZP4*. Therefore, these miRNAs and genes were in high abundances in high- versus low-fertility capacitated sperm. Further, gene *ALB* was linked to fertilization, *SPAM1* linked to sperm penetration through cumulus matrix, and *PAEP, ZP3,* and *ZP4* linked to sperm–zona pellucida binding. The presence of these miRNAs and genes in capacitated sperm populations implied that they were needed for zona binding and fertilization.

Ejaculates or sperm populations with a high proportion of spontaneous AR result in poor fertilization [85,86]. However, sperm have mechanisms that protect them from undergoing spontaneous AR. For example, Ca2 +/calmodulin-dependent protein kinase II (CaMKII) protected mouse sperm from undergoing spontaneous AR by interacting with multi-PDZ domain protein 1 [87]. Inhibition of CaMKII in bovine sperm during incubation under capacitation conditions strongly induces spontaneous AR [24]. In the current study, although the pre-capacitated sperm population in the high-fertility group had greater abundance of *CALM1* and *CALM3* compared to post-capacitated sperm, abundances did not differ between post-capacitated and acrosome-reacted sperm populations, implicating calmodulin in sperm function. A sustained rise in CaMKII activity triggered egg-activation events, including cell cycle resumption, and degradation and recruitment of maternal mRNAs. Sperm entry into an oocyte causes persistent oscillations of intracellular Ca^2+^ in the ooplasm, whereas Ca^2+^ release is the common signal of oocyte activation [88]. Although the Ca^2+^ oscillation mechanism and its relationship with the completion of meiosis are unknown, it involves other proteins, e.g., CAMK-II, cyclin, and cohesin, that hold chromosomes together, and inactivation of the mitogen-activated protein (MAP)-kinase, involved in increased DNA synthesis. All these events are critical for a downstream signaling cascade modulated by Ca^2+^ release [89,90,91]. The specific frequency of Ca^2+^ spikes can affect oocyte activation and early embryo development, resulting in fewer pregnancies [92].

In humans, miR-191 expression was higher in subjects with high rates of fertilization and high-quality embryo production after IVF than in those with low fertilization rates and low-quality embryos, implicating hsa-mir-191-5p in fertilization [93]. Furthermore, a correlation between miR-191 and sperm morphology implied that mir-191could have a key role in maintaining normal sperm morphology. We reported [94] that miR-191 expressions in sperm and seminal plasma were lower in high- compared to low-fertility bulls. However, in the present study, expression of miR-191 in capacitated sperm was not different between high- and low-fertility bulls (1.6 vs. 1.0-fold; *p* > 0.1), although expression of miR-191 in capacitated sperm was lower in abundance in low-fertility bulls in relation to high-fertility bulls (0.6 vs. 1.0-fold).

In the current study, miR-34c and miR-449 were upregulated in high-fertility capacitated sperm. In double knockout mice, miR-34c and miR-449 clusters were important for spermatogenesis and fertility, but not for fertilization nor preimplantation embryo development [95]. Further, in individual miRNA studies, sperm-borne miR-34c is required for the first cell division in mouse embryos [96]. These apparently conflicting results may have been due to off-target effects of the inhibitor of miR-34c used. Regardless, these sperm-borne mRNAs are engaged in acrosome function and ZP binding, and potentially involved in fertilization and embryonic development.

## 5. Conclusions

In conclusion, differentially expressed miRNAs and associated genes elucidated in capacitated sperm of high- versus low-fertility bulls in this study regulated critical pathways that are specific to acrosome function and zona binding. Thus, highly differentially expressed miRNAs in bovine sperm have potential for predicting sperm acrosome functions and fertility.

## Figures and Tables

**Table 1:** Differentially expressed miRNAs, and their associated hub genes and linked reproductive functions in highly fertile sperm in bulls.

Differentally Expressed miRNAs	Hub Genes	Sperm Functions	Organ	References
miR-107	*ALB*	Acrosome reaction, fertilization	Testis, seminal vesicle, prostate, epididymis, sperm	[42,43,44]
miR-107	*FDXR*	Cholesterol efflux, oxidative stress	Testis, seminal vesicle, prostate, sperm	[45,46,47]
miR-125a	*PGR*	Acrosome reaction, capacitation	Testis, prostate, sperm	[48]
miR-129a-5p, miR-133b, miR-17-5p, miR-193a-3p, miR-320a	*CALM1*	Sperm motility, capacitation, acrosome reaction	Testis, seminal vesicle, prostate, sperm	[49,50,51]
miR-129-5p	*RAB27A*	Acrosome reaction	Sperm	[52,53]
miR-137, miR-296-5p, miR-320a, miR-34c, miR-375	*CALM3*	Sperm motility, capacitation, acrosome reaction	Testis, sperm	[54,55]
miR-17-5p, miR-217, miR-421	*CD46*	Acrosome reaction	Sperm	[56,57,58]
miR-206	*CALM2*	Acrosome reaction	Testis, sperm	[55,59]
miR-23b	*ZP4*	Spermatozoa–zona pellucida binding	Testis, prostate, sperm	[60,61]
miR-27a	*PAEP (Glycodelin)*	Motility, spermatozoa–zona pellucida binding	Testis, seminal vesicle, prostate, seminal plasma	[62]
miR-320a, mir-326, miR-330	*ACR*	Motility, acrosome reaction, capacitation	Testis, sperm	[63,64,65]
miR-330, miR-421	*FDXR*	Sperm DNA and mitochondria function, acrosome reaction	Testis, seminal vesicle, prostate, sperm	[66,67]
miR-34c, miR-375, miR-449a	*AREG*	Ion transport, sperm concentration, male system development	Testis	[68]
miR-34c, miR-449a	*SPAM1*	Sperm maturation, sperm penetration through cumulus matrix	Testis, prostate, epididymis, sperm	[69,70,71]
miR-7	*ZP3*	Acrosome reaction, sperm–oocyte binding	Testis, prostate, sperm	[72,73,74]

## Data Availability

The original contributions presented in the study are included in the article/Supplementary Materials; further inquiries can be directed to the corresponding author.

## Supplementary Materials

The following supporting information can be downloaded at: https://www.mdpi.com/article/10.3390/ani14060833/s1. Table S1. Bovine miRBase profiler plate, consisting of prioritized miRNAs and control genes. Table S2. Forward and reverse primer sequences for quantitative real-time polymerase chain reaction amplification of mRNA for selected genes. Table S3. Nucleotide sequences of human and cattle miRNAs (www.miRBase.org, accessed on 26 June 2023). Figure S1. The ethidium bromide-stained electrophoresis gel, with amplicons of expected sizes. Supplementary File S1: Predicted integrated genes of bovine-specific upregulated miRNAs of high- compared to low-fertility Holstein bull sperm (http://www.mirnet.ca/, accessed on 26 June 2023). Supplementary File S2: Biological process gene ontology (GO) functional annotation terms and Kyoto Encyclopedia of Genes and Genomes (KEGG) enrichment pathways for integrated genes of upregulated miRNAs of high-fertility Holstein bull sperm. Supplementary File S3: Gene ontology (GO) terms for predicted/hub genes associated functional terms (http://www.ici.upmc.fr/cluego.shtml, accessed on 26 June 2023).
