# Supplementary material for: MicroRNAs and Their Associated Genes Regulating the Acrosome Reaction in Sperm of High- versus Low-Fertility Holstein Bulls
Source: Animals (Basel). 2024 Mar 8;14(6):833. doi: 10.3390/ani14060833 (PMC10967381; doi:10.3390/ani14060833)
Supplement: Supplementary file 1 [file animals-14-00833-s001.zip › Figure S1.pdf]

Figure S1. The ethidium bromide-stained electrophoresis gel, with amplicons of expected sizes

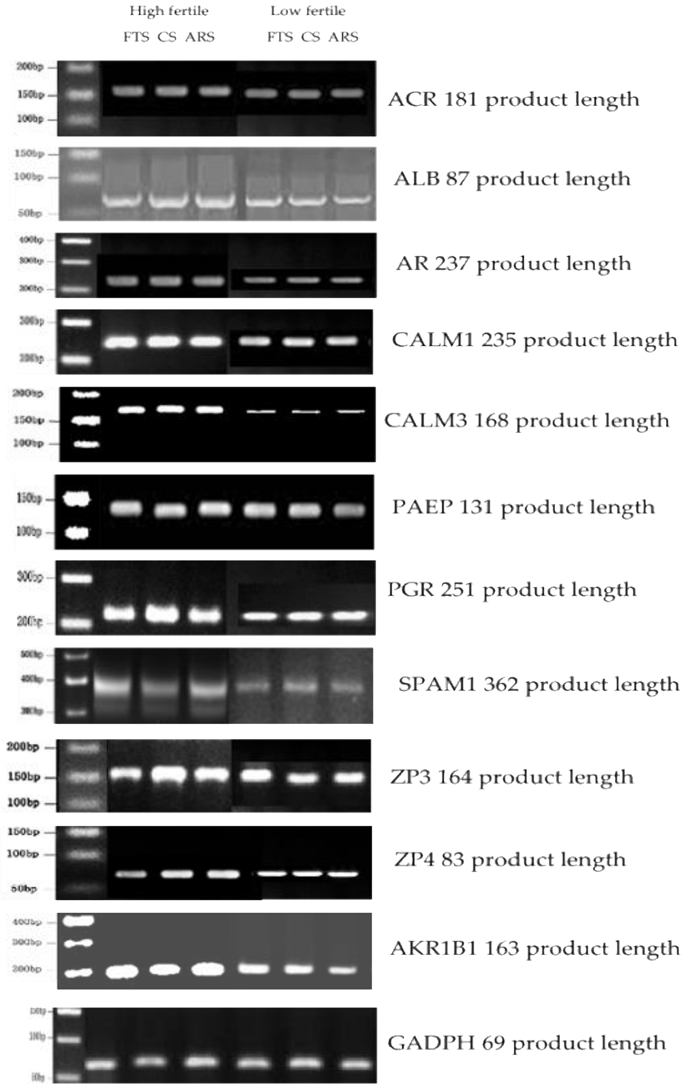

FTS, Frozen-thawed sperm; CS, capacitated sperm; ARS, Acrosome induced sperm;
ACR, Acrosin; ALB, Albumin; AR, Androgen Receptor; CALM, Calmodulin; PAEP, Progestogen Associated Endometrial Protein; PGR, Progesterone Receptor; SPAM, Sperm Adhesion Molecule; ZP, Zona Pellucida Glycoprotein; GADPH, Glyceraldehyde-3-phosphate dehydrogenase.
