# Supplementary material for: MicroRNAs and Their Associated Genes Regulating the Acrosome Reaction in Sperm of High- versus Low-Fertility Holstein Bulls
Source: Animals (Basel). 2024 Mar 8;14(6):833. doi: 10.3390/ani14060833 (PMC10967381; doi:10.3390/ani14060833)
Supplement: Supplementary file 1 [file animals-14-00833-s001.zip › Table S1.pdf]

Table S1. | Bovine miRBase profiler plate, consisting of primers for target miRNAs and control genes.

Layout	1	2	3	4	5	6	7	8	9	10	11	12
**A**	bta-let-7f	bta-miR-101	bta-miR-103	bta-miR-125a	bta-miR-125b	bta-miR-126-3p	bta-miR-128	bta-miR-145	bta-miR-148a	bta-miR-151-3p	bta-miR-151-5p	bta-miR-16b
**B**	bta-miR-181a	bta-miR-18a	bta-miR-18b	bta-miR-199a-5p	bta-miR-206	bta-miR-20a	bta-miR-21-5p	bta-miR-221	bta-miR-26a	bta-miR-26b	bta-miR-27a	bta-miR-29a
**C**	bta-miR-31	bta-miR-320a	bta-miR-326	bta-miR-330	bta-miR-34c	bta-miR-375	bta-miR-421	bta-miR-499a	bta-miR-99a-5p	bta-miR-7	bta-miR-7a-5p	bta-let-7d
**D**	bta- let-7g	bta- let-7i	bta-miR-106a	bta-miR-107	bta-miR-10a	bta-miR-10b	bta-miR-122	bta-miR-124b	bta-miR-129-5p	bta-miR-133b	bta-miR-137	bta-miR-139
**E**	bta-miR-140	bta-miR-142-3p	bta-miR-142-5p	bta-miR-148b	bta-miR-150	bta-miR-15b	bta-miR-17-3p	bta-miR-17-5p	bta-miR-181b	bta-miR-181c	bta-miR-186	bta-miR-191
**F**	bta-miR-192	bta-miR-193a-3p	bta-miR-193a-5p	bta-miR-199a-3p	bta-miR-199b	bta-miR-200a	bta-miR-200b	bta-miR-200c	bta-miR-20b	bta-miR-210	bta-miR-214	bta-miR-215
**G**	bta-miR-217	bta-miR-218	bta-miR-22-5p	bta-miR-23a	bta-miR-23b	bta-miR-24-3p	bta-miR-25	bta-miR-296-5p	bta-miR-30a-5p	bta-miR-30b-5p	bta-miR-30c	bta-miR-30e-5p
**H**	cel-miR39-3p	cel-miR39-3p	SNORD42B	SNORD69	SNORD61	SNORD68	SNORD96A	RNU6-6P	miRTC	miRTC	PPC	PPC

Characterized target miRNAs (plate well positions A1 – G12) and controls (plate well positions H1 – H12).
