# Supplementary material for: MicroRNAs and Their Associated Genes Regulating the Acrosome Reaction in Sperm of High- versus Low-Fertility Holstein Bulls
Source: Animals (Basel). 2024 Mar 8;14(6):833. doi: 10.3390/ani14060833 (PMC10967381; doi:10.3390/ani14060833)
Supplement: Supplementary file 1 [file animals-14-00833-s001.zip › Table S2.pdf]

Table S2. | Forward and reverse primer sequence for quantitative real-time polymerase chain reaction amplification of mRNA for bovine sperm samples.

| Gene | Forward Primer | Reverse primer | Product length | Accession number |
|---|---|---|---|---|
| ACR | CCCTGTGGGACACGGTTC | TCGGCTTCACTGGCTTATTGC | 181 | NM_173886.2 |
| ALB | GCTGTGGAGGGTCCAAAACT | TCAGGTAGGCTGAGATGCCT | 87 | NM_180992.2 |
| AR | ACCTGTGTGCCAGCAGAAAT | TGGGCTGACACTCATAGCCT | 237 | NM_001244127.1 |
| CALM1 | CAGAGCCGTTAGCGCAGTTG | TGCCATCACCGTCTTTGTCA | 235 | NM_001242572.1 |
| CALM3 | CCCCTCAGGTCGATCAAGTG | CCGAAAACGTCCACAGCTTC | 168 | NM_001046249.2 |
| PAEP | CACATCTAGGTGAGCCCCTG | CAGGAGGGGTGAATGTGGTC | 131 | NM_173929.3 |
| PGR | TACCTTAGGCCGGATTCAGA | ACTTTCTAAGGCGACATGCTGG | 251 | NM_001205356.1 |
| SPAM1 | ACGGGCAGAGATGACAAGATGA | CATTCCAGGCCCAGAGGAAAG | 362 | NM_001008413.3 |
| ZP3 | GTCGATGCTGTAGCAAGGG | ACGCCACGGTCATTCATCTT | 164 | NM_173974.3 |
| ZP4 | TCCTGTGAATCTCCCAGATGT | GTGAAGGAGCACATGGCAGT | 83 | NM_173975.2 |
| AKR1B1 | CAAGCAGTCATGGCCAACCA | CATTCTGGTACACGTGGGCA | 163 | NM_001012519.1 |
| GAPDH | GTGAAGGTCGGAGTGAACGG | ATTGATGGCGACGATGTCCA | 93 | NM_001034034.2 |

ACR, Acrosin; ALB, Albumin; AR, Androgen Receptor; CALM, Calmodulin; PAEP, Progestogen Associated Endometrial Protein; PGR, Progesterone Receptor; SPAM, Sperm Adhesion Molecule; ZP, Zona Pellucida Glycoprotein; GADPH, Glyceraldehyde-3-phosphate dehydrogenase.
