# Supplementary material for: MicroRNAs and Their Associated Genes Regulating the Acrosome Reaction in Sperm of High- versus Low-Fertility Holstein Bulls
Source: Animals (Basel). 2024 Mar 8;14(6):833. doi: 10.3390/ani14060833 (PMC10967381; doi:10.3390/ani14060833)
Supplement: Supplementary file 1 [file animals-14-00833-s001.zip › Table S3.pdf]

Table S3. | Human and bovine miR nucleotide sequences.

miR-id	Human Sequence	Bovine Sequence
bta-miR-107	AGCAGCAUUGUACAGGGCUAUCA	AGCAGCAUUGUACAGGGCUAUC
bta-mir-125a	UCCCUGAGACCCUUUAACCUGUGA	UCCCUGAGACCCUUUAACCUGUG
bta-mir-129-5p	CUUUUUGCGGUCUGGGCUUGC	CUUUUUGCGGUCUGGGCUUGCU
bta-mir-133b	UUUGGUCCCCUUCAACCAGCUA	UUUGGUCCCCUUCAACCAGCUA
bta-mir-137	UUAUUGCUUAAGAAUACGCGUAG	UUAUUGCUUAAGAAUACGCGUAG
bta-mir-17-5p	CAAAGUGCUUACAGUGCAGGUAG	CAAAGUGCUUACAGUGCAGGUAGU
bta-mir-193a-3p	AACUGGCCUACAAAGUCCCAGU	AACUGGCCUACAAAGUCCCAGU
bta-mir-206	UGGAAUGUAAGGAAGUGUGUGG	UGGAAUGUAAGGAAGUGUGUGG
bta-mir-217	UACUGCAUCAGGAACUGAUUGGA	UACUGCAUCAGGAACUGAUUGGAU
bta-mir-23b	AUCACAUUGCCAGGGAUUACCAC	AUCACAUUGCCAGGGAUUACCAC
bta-mir-27a	UUCACAGUGGCUAAGUUCCGC	UUCACAGUGGCUAAGUUCCG
bta-mir-296-5p	AGGGCCCCCCCUCAAUCCUGU	GAGGGCCCCCCCCAAUCCU
bta-mir-320a	AAAAGCUGGGUUGAGAGGGCGA	AAAAGCUGGGUUGAGAGGGCGA
bta-mir-326	CCUCUGGGCCCUUCCUCCAG	CCUCUGGGCCCUUCCUCCAG
bta-mir-330	GCAAAGCACACGGCCUGCAGAGA	GCAAAGCACACGGCCUGCAGAGA
bta-mir-34c	AGGCAGUGUAGUUAGCUGAUUGC	AGGCAGUGUAGUUAGCUGAUUG
bta-mir-375	UUUGUUCGUUCGGCUCGCGUGA	UUUUGUUCGUUCGGCUCGCGUGA
bta-mir-421	AUCAACAGACAUUAAUUGGGCGC	AUCAACAGACAUUAAUUGGGCGC
bta-mir-7	UGGAAGACUAGUGAUUUUGUUGUU	UGGAAGACUAGUGAUUUUGUUGUU
